# Exploring the lived experiences of women with breast cancer across the care continuum: a systematic meta-aggregative qualitative review protocol

**DOI:** 10.1136/bmjopen-2026-120045

**Published:** 2026-10-20

**Authors:** Seemela Desiree Malope, Maureen Joffe, Shane Norris, Charmaine Blanchard, Ngcwalisa Amanda Jama, Anri Smit, Kelechi Elizabeth Oladimeji

**Affiliations:** 1 SAMRC/Wits Developmental Pathways for Health Research Unit, Faculty of Health Sciences, University of the Witwatersrand Johannesburg, Johannesburg, GP, South Africa; 2 Network for Oncology Research in Africa (NORA), Global Health Working Group, Martin-Luther-Universitat Halle-Wittenberg, Halle (Saale), Germany; 3 School of Human Development and Health, University of Southampton, Southampton, England, UK

**Keywords:** Cancer Pain, Cancer Survivors, Health Care Costs, Health Education, Health Services

## Abstract

**Introduction:**

Breast cancer is the most commonly diagnosed cancer among women globally and remains a leading cause of cancer-related mortality. Although epidemiological trends and clinical outcomes are well documented, less consolidated evidence exists regarding how women experience diagnosis, treatment, survivorship and health system navigation, particularly in resource-constrained settings. Since the publication of a 2019 meta-synthesis of women’s narratives of breast cancer, qualitative research in this field has expanded substantially. An updated synthesis is therefore warranted. Hence, this systematic qualitative review aims to synthesise updated evidence on the lived experiences of women diagnosed with breast cancer across the care trajectory and explore how these experiences may inform patient-centred clinical practice and health policy in diverse global contexts.

**Methods and analysis:**

This review will update prior evidence by synthesising studies published between 1 February 2018 and 31 January 2026. The review will be conducted in accordance with the Joanna Briggs Institute (JBI) meta-aggregation methodology. The review question is framed using the Population, Phenomenon of Interest and Context framework, while eligibility criteria are operationalised using a modified framework to reflect the qualitative focus of the review. The protocol has been developed in line with the Preferred Reporting Items for Systematic Review and Meta-Analysis Protocols (PRISMA-P) guidelines. Electronic databases including PubMed, Scopus, Web of Science and EBSCOhost/CINAHL will be searched. Eligible studies will include peer-reviewed qualitative research reporting first-person accounts of women assigned female sex at birth, reflecting female biological sex and aged ≥18 years, diagnosed with breast cancer. Screening, data extraction and methodological appraisal will be undertaken independently by multiple reviewers. Methodological quality will be assessed using the JBI Critical Appraisal Checklist for qualitative research, findings will be synthesised using the JBI meta-aggregation approach and confidence in the evidence will be evaluated using ConQual.

**Ethics and dissemination:**

As this review uses published literature only, ethical approval is not required. Findings will be disseminated through peer-reviewed publication, conference presentations and stakeholder engagement to inform patient-centred oncology care and health system strengthening globally.

**Trial registration number:**

The protocol is registered on the Open Science Framework. The registered protocol is available at https://osf.io/wgzck/ (Registration DOI - 10.17605/OSF.IO/WGZCK).

**STRENGTHS AND LIMITATIONS OF THIS STUDY**

- The review updates and extends the 2019 meta-synthesis by incorporating qualitative evidence published between February 2018 and January 2026 and applying the Joanna Briggs Institute (JBI) meta-aggregation approach to support a transparent and practice-oriented synthesis.
- Independent screening, data extraction and critical appraisal by multiple reviewers will enhance methodological rigour and reduce the risk of interpretive bias, with confidence in the synthesised findings assessed using the ConQual approach.
- Restriction to English-language, peer-reviewed publications and exclusion of grey literature, dissertations and theses may result in the omission of relevant evidence and increase the risk of publication and language bias.
- Although the review protocol was prospectively registered on the Open Science Framework, it was not registered in International Prospective Register of Systematic Reviews (PROSPERO), which may reduce its visibility among researchers using PROSPERO to identify registered systematic reviews.
- Use of meta-aggregation as the sole qualitative synthesis approach may limit opportunities for interpretive or critical synthesis, theory generation and deeper exploration of contextual and explanatory insights.

## Introduction

 Cancer remains a leading cause of global morbidity and mortality, with incidence projected to rise substantially over the coming decades.^1 2^ Breast cancer is the most commonly diagnosed cancer among women worldwide, accounting for approximately 11%–12% of all new cancer cases and nearly one in four cancer diagnoses.^3 4^ Despite advances in detection and treatment, breast cancer continues to impose a significant burden, particularly in developing countries, where late presentation, limited access to care and health system constraints contribute to poorer outcomes.^5 6^ Experiencing a chronic disease such as cancer forces affected individuals to consider the burden of emotional, financial, medical, social and cultural demands that usually accompany such a diagnosis.^7^ A substantial body of literature documents the risk factors, clinical features, treatment options and physical sequelae of breast cancer.^8–12^ Breast cancer is not only a biomedical condition but also a deeply embodied and socially situated illness.^13 14^ In many cultural contexts, breasts are symbolically associated with femininity, sexuality, motherhood and identity.^15 16^ Consequently, the physical consequences of treatment such as mastectomy, scarring and chemotherapy-induced alopecia can have profound implications for body image, self-perception, intimate relationships and social participation.^17 18^ These visible and lasting changes often extend beyond physical discomfort to encompass psychological distress, altered self-esteem and disruptions to social and cultural roles.^19–22^ The lived experience of breast cancer therefore involves far more than symptom burden; it reflects the intersection of illness with identity, relationships and everyday life.

Qualitative research provides insight into how individuals interpret illness, navigate treatment decisions and construct meaning across the breast cancer care continuum.^23^ Through in-depth engagement with participants’ accounts, qualitative inquiry captures how women make sense of the pre-diagnostic phase, navigate the processes leading to a definitive cancer diagnosis, cope with uncertainty and respond to shifting bodily and social realities through treatment and survivorship. Synthesising such evidence allows for the identification of recurring experiential patterns while preserving contextual nuance and cultural specificity.^24^ In 2019, Smit *et al*^19^ conducted a systematic review and meta-synthesis of women’s narratives of living with breast cancer, generating the ‘Breast Cancer Trajectory’ framework. This framework described experiential domains across pre-diagnosis, diagnosis, treatment, survivorship, recurrence and advanced disease stages. While this synthesis provided a valuable conceptual foundation, the qualitative literature has expanded considerably since its publication, including studies conducted in diverse socio-economic and health system contexts. Despite this growth, no updated synthesis has systematically integrated the emerging body of evidence. The present review therefore updates and extends the prior synthesis, with a specific focus on women’s lived experiences across the breast cancer care continuum, spanning symptom recognition and appraisal, diagnostic processes, treatment and survivorship. It applies the Joanna Briggs Institute (JBI) meta-aggregation approach to synthesise qualitative evidence that aggregates findings from primary studies into categories and synthesised findings without re-interpretating them. It emphasises methodological transparency, structured credibility assessment and actionable outputs.^24^ This updated synthesis aims to strengthen the evidence base informing equitable and contextually responsive breast cancer care globally. Findings may guide policy and practice for feasible and affordable interventions to address breast cancer patient needs across the breast cancer care continuum.

### Review question

Guided by the Population, Interest, and Context (PICo) framework of JBI, this review seeks to address the following primary question: what are women’s lived experiences with breast cancer across the care continuum? As a secondary analytic objective, the review will explore similarities and differences in these experiences across high-income country (HIC) and low-income and middle-income country (LMIC) contexts where supported by the included evidence.

PICo was used to frame the review question, while a modified sample, phenomenon of interest, design, evaluation and research type (SPIDER) framework was subsequently used to operationalise the eligibility criteria for this qualitative evidence synthesis.

## Methods

The review will follow the JBI methodology for systematic reviews of qualitative evidence and meta-aggregation.^24 25^ Meta-aggregation is informed by two complementary philosophical traditions: Husserlian transcendental phenomenology and pragmatism.^25 26^ Phenomenology emphasises understanding the intentionality and meaning of individuals’ lived experiences, focusing on how participants perceive and interpret the phenomena they encounter. Pragmatism, in contrast, prioritises the practical application of knowledge and the generation of findings that can inform healthcare decision-making. Within this approach, findings from primary qualitative studies are systematically extracted, grouped according to similarity of meaning and synthesised into higher-order findings. This process preserves the meanings expressed in the original studies while generating actionable knowledge that can inform recommendations for clinical practice, health policy and future research.

The review protocol will adhere to the Preferred Reporting Items for Systematic Review and Meta-Analysis Protocols (PRISMA-P) statement, and the final report will be prepared following the PRISMA 2020 guidelines.^27^ The review will update and extend a prior meta-synthesis by Smit *et al*,^19^ incorporating evidence published from 1 February 2018 through 31 January 2026. The review was initially intended for registration with PROSPERO. However, because database searches and initial screening had already commenced at the time of registration enquiry, the protocol no longer meets PROSPERO eligibility criteria. It was therefore registered on the Open Science Framework (OSF). The OSF registration (DOI-10.17605/OSF.IO/WGZCK-https://osf.io/wgzck/) provides a time-stamped record of the methodology and update process; however, the absence of pre-search PROSPERO registration is acknowledged as a limitation in transparency.

### Eligibility criteria

Eligibility criteria were operationalised a priori using a modified SPIDER framework to ensure conceptual clarity and reproducibility.^28 29^ While PICo was used to frame the overarching review question, SPIDER was used to translate the question into explicit eligibility criteria for this qualitative evidence synthesis, particularly by specifying the sample, phenomenon of interest, eligible qualitative study designs, experiential data of interest and research type. The two frameworks were therefore applied at different stages of the review process and were not used interchangeably.

### Inclusion criteria

#### Participants (sample)

Studies included will be those reporting on women assigned female at birth (AFAB), reflecting female biological sex and aged ≥18 years who have been diagnosed with breast cancer including breast cancer recurrence. Studies including mixed-gender samples will be included only if findings specific to women as earlier defined are extractable.

#### Phenomenon of interest (evaluation)

The review focuses on women’s lived experiences of breast cancer. This includes experiential accounts related to the pre-diagnostic phase, diagnosis, treatment decision-making, treatment experiences, psychosocial adjustment, survivorship, recurrence/advanced disease and navigation of healthcare systems. Studies must present first-person experiential data derived from qualitative inquiry, spanning feelings, attitudes, beliefs, behaviours and perceptions. Experiences may include challenges, enablers, needs, support, adjustment, coping, positive and negative changes and benefits.

#### Context

Studies conducted in any global geographical setting will be eligible for consideration. Studies across the world will be included to enable global contextual comparison, including comparison of findings from HIC and LMIC contexts.

#### Study design (research type)

Eligible studies include primary qualitative research employing methodologies such as phenomenology, grounded theory, ethnography, narrative inquiry, qualitative descriptive design or other recognised qualitative approaches. Mixed-methods studies will be included where qualitative findings are clearly identifiable and extractable.

#### Publication characteristics

Studies must:

1. Be published in peer-reviewed journals.
2. Be written in English.
3. Have been published between 1 February 2018 and 31 January 2026.

### Exclusion criteria

Studies will be excluded if they:

1. Are quantitative only.
2. Are systematic reviews, scoping reviews, narrative reviews or meta-analyses.
3. Are conference abstracts, editorials, commentaries, policy briefs, dissertations or theses.
4. Feasibility and/or acceptability studies, trial-based studies, intervention and evaluation studies.
5. Focus exclusively on healthcare providers’, caregivers’, or family members’ perspectives without extractable patient perspective data.
6. Are studies where the study population includes transgender individuals that were not AFAB and not of female biological sex at birth.
7. Were published prior to 1 February 2018

The search commenced on 1 February 2018, immediately following the January 2018 search end date of the previous systematic review by Smit et al.^19^ Thereby ensuring continuity of the evidence base while avoiding overlap in study inclusion.

### Ethics and dissemination statement

#### Ethics approval

Ethical approval was not sought because this study is a systematic review and synthesis of data from previously published studies. No new data were collected from human participants, and no individual patient-level or identifiable data were accessed. Findings will be disseminated through peer-reviewed publication, conference presentations and stakeholder engagement to inform patient-centred oncology care and health system strengthening globally.

#### Data availability statement

Data are available on reasonable request. Requests should be directed to the corresponding author.

### Patient and public involvement

Patients and/or the public were not involved in the design, conduct, reporting or dissemination plans of this research.

## Search methods

### Information sources

A comprehensive electronic database search will be conducted across multidisciplinary databases covering oncology, nursing, psychology and social sciences. The following databases will be searched: Web of Science, Scopus, PubMed and EBSCOhost/CINAHL platforms.

### Search strategy

A three-step search strategy will be employed to identify relevant qualitative studies addressing the review question. First, an initial limited search of selected databases will be undertaken to identify relevant articles. The text words contained in the titles and abstracts of relevant articles, together with the index terms used to describe them, will be analysed to develop a comprehensive search strategy.

Second, the identified keywords and subject headings will be used to construct database-specific search strategies. Controlled vocabulary terms and free-text keywords will be combined, and search strings adapted to the indexing structure and syntax of each database. The search strategy will be developed by the review team in consultation with an academic librarian, who will provide guidance on database selection, keyword development, controlled vocabulary terms and search syntax to enhance the sensitivity and comprehensiveness of the search. Search strings will be database-specific rather than identical but will retain the same core concepts across databases. Core search concepts will include the following:

1. Breast cancer.
2. Lived experience/experiential accounts.
3. Qualitative research methods.

Lastly, we will manually perform a reference list checking of all included studies in Smit *et al*.^19^ A systematic review will be performed to identify any additional potentially eligible articles, consistent with the third stage of the JBI qualitative evidence synthesis search strategy.^24^

An example search structure also available in online supplemental file 1 is as follows:

(“breast cancer” OR “breast neoplasm*”) AND (“lived experience*” OR “experience*” OR “narrative*” OR “perception*” OR “meaning-making”) AND (“qualitative” OR “interview*” OR “focus group*” OR “ethnography*” OR “phenomenology*” OR “grounded theory”)

### Search validation

Search validation will be undertaken through:

1. Pilot searches to confirm retrieval of known key qualitative studies in the field.
2. Iterative refinement of keywords and subject headings.
3. Cross-checking indexing terms against included studies.

### Study selection

All search results will be exported in research information systems (RIS) format and imported into EndNote V.20 (Clarivate Analytics, PA, USA), for automatic and manual deduplication. Deduplicated records will then be uploaded into Covidence systematic review software, Veritas Health Innovation, Melbourne, Australia. Available at www.covidence.org and Microsoft Excel 2019 (Redmond, WA, USA) for screening of titles and abstracts.

### Screening will proceed in two stages

#### Stage 1: title and abstract screening

Two reviewers (KEO and SDM) will independently screen titles and abstracts against predefined eligibility criteria. Studies will be advanced to full-text review if deemed potentially eligible by at least one reviewer to minimise the risk of erroneous exclusion. Discrepancies will be discussed between the reviewers, with a third reviewer (NJ and MJ) consulted where necessary.

#### Stage 2: full-text screening

Full-text articles identified as potentially eligible during title and abstract screening will be retrieved and independently assessed by two reviewers (KEO and SDM) against the predefined eligibility criteria. Screening decisions and reasons for exclusion will be documented using Covidence systematic review software and recorded in Microsoft Excel. Disagreements at both screening stages will be resolved through discussion and consensus. Where consensus cannot be reached, third reviewers (NJ and MJ) will adjudicate. The study selection process will be reported using a PRISMA flow diagram, documenting the number of records identified, screened, excluded with reasons and included in the review.

### Quality appraisal

Methodological quality of included studies will be assessed using the JBI critical appraisal checklist for qualitative research.^24^ The checklist evaluates congruity between the research aims, methodology, data collection, analysis, interpretation and representation of participants’ voices, as well as attention to researcher reflexivity. Eligible studies will be critically appraised independently by two reviewers (KEO and SDM). A pilot appraisal will be conducted on one study to ensure a shared understanding of the checklist criteria before the remaining studies are assessed independently. The JBI critical appraisal checklist comprises 10 questions with four response options (yes, no, unclear or not applicable). Where clarification of methodological details is required, study authors may be contacted by email to request additional information. Disagreements between reviewers will be resolved through discussion and consensus, with adjudication by third reviewers (NJ and MJ) where necessary. Studies will not be excluded solely on the basis of methodological quality. Instead, the outcomes of the quality appraisal will inform the assessment of confidence in the synthesised findings using the ConQual approach.

### Data extraction

Data extraction will be undertaken independently by two reviewers (KEO and SDM) using a structured data extraction form developed and piloted within Covidence. Following extraction, data will be exported to a standardised Microsoft Excel spreadsheet for further data management, verification and analysis. The data extraction form will be refined, where necessary, following pilot testing to ensure consistency, completeness and applicability across the included studies. Any discrepancies between reviewers will be resolved through discussion and, where required, consultation with a third reviewer. Data extraction in meta-aggregation occurs in two phases. In the first phase, study characteristics will be extracted, including bibliographic details (author, year of publication and journal), country and study setting, study aims, qualitative methodology, participant characteristics and data collection methods. Where reported, details such as timing and duration of interviews will also be captured. In the second phase, qualitative findings and their supporting illustrations will be extracted verbatim as reported by the study authors. In accordance with JBI meta-aggregation guidance, findings will be linked to illustrative participant quotations and assigned a level of credibility (unequivocal, credible or unsupported). Disagreements between reviewers will be resolved through discussion and consensus. Extracted findings will first be classified according to their credibility (unequivocal, equivocal or unsupported) based on the presence and clarity of supporting illustrations. In the context of JBI meta-aggregation, credibility refers to the extent to which a reported finding is supported by clear and explicit evidence from the primary study, typically in the form of illustrative participant quotations or field data. Unequivocal findings are accompanied by clear participant evidence that leaves little room for alternative interpretation, equivocal findings are supported by data but remain open to interpretation, and unsupported findings lack sufficient illustrative evidence. Unsupported findings will be excluded from aggregation in accordance with JBI guidance. Where consensus cannot be reached, third reviewers (NJ and MJ) will adjudicate. Where necessary, study authors may be contacted by email to request clarification or additional information to facilitate data extraction and synthesis.

### Data synthesis

Qualitative findings will be synthesised using the JBI meta-aggregation approach. Credible findings will be grouped into categories based on similarity of meaning, and these categories will subsequently be aggregated into synthesised findings representing overarching experiential domains across the breast cancer care continuum. Synthesis decisions will be undertaken collaboratively by KEO and SDM. Divergences in categorisation or aggregation will be resolved through structured discussion, with adjudication by NJ where required. Where textual pooling is not possible, findings will be presented in narrative form.

### Assessing confidence in synthesised findings (ConQual)

Confidence in each synthesised finding will be assessed using the ConQual approach, incorporating assessments of dependability (informed by JBI critical appraisal outcomes) and credibility (informed by classification of findings). In this context, dependability refers to the methodological consistency and reliability of the primary studies, as reflected in the congruity between research aims, methodology, data collection, analysis and interpretation and will be informed by the outcomes of the JBI critical appraisal. ConQual assessments will be undertaken by KEO and SDM, with discrepancies resolved through discussion and consultation with NJ where necessary.

Dependability will be informed by the JBI critical appraisal outcomes of the contributing studies, particularly methodological congruity between the research question, methodology, data collection, analysis and interpretation, representation of participants’ voices and consideration of researcher reflexivity. Synthesised findings will be considered for downgrading where contributing studies show important methodological limitations across these domains. Credibility will be informed by the classification of contributing findings as unequivocal, credible or unsupported. Unsupported findings will be excluded from aggregation. Synthesised findings supported mainly by unequivocal findings will retain stronger credibility, whereas those relying substantially on credible findings that are open to interpretation will be considered for downgrading.

Downgrading decisions and supporting justifications will be reviewed with members of the wider review team, including MJ, CB and SN where necessary. Any unresolved disagreements will be resolved through discussion with the review team. A ConQual summary of findings table will be produced to enhance transparency. This table will report the review context (population, phenomenon of interest and setting), each synthesised finding, the contributing studies, assessments of dependability and credibility, and the resulting overall ConQual score.

### Assessment of meta-bias

Formal statistical assessment of publication bias is not applicable to qualitative evidence synthesis. The review is restricted to peer-reviewed publications, which may introduce publication bias; this limitation will be acknowledged. Selective reporting will be considered during quality appraisal by examining congruence between study objectives and reported findings. KEO and SDM will assess whether certain experiential domains appear under-represented, with discrepancies discussed and adjudicated by NJ if required. The possibility of missing voices or under-reported perspectives will be addressed in the interpretation of findings.

### Status of the review

At the time of manuscript submission, the review is at the screening stage. Title and abstract screening of retrieved records is ongoing, with full-text assessment to follow in accordance with the predefined protocol. About 11 649 records were identified from four databases. After removal of 6276 duplicates, 5373 records were screened by title and abstract. Of these, 529 full-text articles were assessed for eligibility (online supplemental file 2, RISMA flow diagram). We anticipate completion of review synthesis and manuscript first draft by 30 September 2026. The final number of included studies and full-text exclusion reasons will be reported in the completed systematic review.

## Supplementary material

10.1136/bmjopen-2026-120045 online supplemental file 1

10.1136/bmjopen-2026-120045 online supplemental file 2
