# Supplementary material for: Exploring the lived experiences of women with breast cancer across the care continuum: a systematic meta-aggregative qualitative review protocol
Source: BMJ Open. 2026 Oct 20;16(10):e120045. doi: 10.1136/bmjopen-2026-120045 (PMC13684198; doi:10.1136/bmjopen-2026-120045)
Supplement: Supplementary file 1 [file bmjopen-2026-120045.reviewer_comments.pdf]

PEER REVIEW HISTORY

BMJ Open publishes all reviews undertaken for accepted manuscripts. Reviewers are asked to complete a checklist review form and are provided with free text boxes to elaborate on their assessment. These free text comments are reproduced below.

ARTICLE DETAILS

Title (Provisional)

Exploring the Lived Experiences of Women with Breast Cancer Across the Care Continuum: A Systematic Meta-Aggregative Qualitative Review Protocol

Authors

Malope, Seemela Desiree; Joffe, Maureen; Norris, Shane; Blanchard, Charmaine; Jama, Ngcwalisa Amanda; Smit, Anri; Oladimeji, Kelechi Elizabeth Elizabeth

---

VERSION 1 - REVIEW

**Reviewer**	**1**
**Name**	**Nweke, Martins**
**Affiliation**	**University of Pretoria, Physiotherapy**
**Date**	**15-Jun-2026**
**COI**	**None**

Thank you for submitting this protocol to BMJ Open. The review addresses a genuinely important question — the lived experiences of women with breast cancer remain underexplored in systematic synthesis since the 2019 Smit et al. meta-synthesis — and the JBI meta-aggregation framework is well-suited to this purpose. The team demonstrates clear methodological competence, and the multi-reviewer design with independent screening, extraction, and appraisal reflects good practice. Revision is required before the protocol can be accepted. Please address the following points in the review document

INTRODUCTION

This manuscript presents a protocol for a systematic meta-aggregative qualitative review of women's lived experiences with breast cancer across the care continuum. The authors aim to update and extend a prior 2019 meta-synthesis by Smit and colleagues, incorporating qualitative evidence published between 1 February 2018 and 31 January 2026. The review is grounded in Joanna Briggs Institute (JBI) methodology, guided by the PICo framework, and the protocol is reported in accordance with PRISMA-P guidelines. The topic is clinically and socially important: breast cancer remains the most common cancer among women globally, and understanding patients' lived experiences is essential for patient-centred care and health system responsiveness, particularly in under-resourced settings.

The protocol is generally well-structured and methodologically sound, reflecting a team with clear familiarity with JBI qualitative synthesis methods. However, several issues ranging from a critical registration concern to gaps in eligibility criteria transparency, search strategy completeness, and language/terminology inconsistencies require attention before this protocol can be accepted for publication.

GENERAL COMMENTS

This is a clearly motivated protocol that fills an acknowledged gap: no updated systematic synthesis of women's breast cancer experiences has been published since the Smit et al. (2019) meta-synthesis. The choice of JBI meta-aggregation is appropriate for a practice-oriented synthesis that aims to generate actionable recommendations. The dual philosophical grounding in Husserlian phenomenology and pragmatism is thoughtfully articulated. The multi-reviewer independent screening and extraction design, the use of Covidence, and the planned ConQual confidence assessment all reflect methodological rigour.

However, the manuscript has a significant transparency issue regarding registration the protocol was not registered with PROSPERO because searching had already begun, and this needs to be addressed more forthrightly. Additionally, the search strategy as documented has notable gaps (only four databases were ultimately searched, despite five or six being listed in the text), the SPIDER/PICo framework duplication is confusing, the gender and sex terminology is applied inconsistently and in places inaccurately, and the PRISMA flow diagram in Supplementary File 2 contains placeholder text that should not appear in a submitted manuscript. These issues are detailed below.

MAJOR COMMENTS

1. The PROSPERO non-registration is a significant transparency concern that requires more direct acknowledgment.

The authors state that the review was initially intended for PROSPERO registration but that searches had already commenced when registration was sought, making the protocol ineligible. This is disclosed which is appreciated but it is presented rather neutrally, without acknowledgment of the implications. PROSPERO registration prior to searching is a fundamental transparency safeguard in systematic review methodology, designed precisely to prevent post-hoc modification of eligibility criteria, outcomes, and analytic decisions based on knowledge of the literature. The fact that searching began before registration was secured represents a protocol deviation that could compromise the credibility of the review. The authors should: (a) explain how and why searching commenced prior to registration; (b) confirm explicitly that eligibility criteria, search strategy, and synthesis approach were finalized before any screening or extraction decisions were made; (c) acknowledge this as a limitation of the protocol's transparency rather than presenting it as a straightforward administrative alternative. Simply registering on OSF does not fully mitigate the loss of the pre-search time-stamp that PROSPERO would have provided.

2. The manuscript describes two different eligibility frameworks (PICo and SPIDER) without adequately reconciling them.

The abstract and introduction state that the review is "guided by the PICo framework (Population, Interest, Context)." The eligibility criteria section then states that "Eligibility criteria were defined a priori using a modified SPIDER framework (Sample, Phenomenon of Interest, Design, Evaluation, Research type)." These two frameworks serve overlapping but distinct purposes, and their simultaneous use without explanation will confuse readers. PICo is appropriate for framing the review question, while SPIDER is more appropriate for operationalising eligibility criteria in qualitative reviews but this distinction is never explained. The authors should clarify how the two frameworks were used at different stages of the review process, or consolidate the framing to use one primary framework consistently throughout.

3. The search strategy is inconsistently described across the manuscript.

The methods section states that the following databases will be searched: "Web of Science, Scopus, PubMed, MEDLINE, and EBSCOhost platform" listing five databases, and earlier the abstract adds "ProQuest" and "ScienceDirect," implying six or seven sources. However, Supplementary File 1 (the actual search table) documents searches in only four databases: PubMed, EBSCOhost/CINAHL, Scopus, and Web of Science with no search conducted in ProQuest, ScienceDirect, or MEDLINE as a separate database. The PRISMA flow diagram confirms that 11,649 records were identified from four databases. This discrepancy between what the text promises and what was actually done must be resolved. If ProQuest and ScienceDirect were dropped, the rationale must be provided. Additionally, the search strings across the four databases are not parallel for example, the Web of Science string omits several experiential terms ("illness perception," "coping strateg*," "emotional impact") that are included in the PubMed and CINAHL strings. This inconsistency should be explained or corrected to ensure sensitivity is equivalent across databases.

4. The gender and sex terminology is inconsistently and in places incorrectly applied.

The manuscript uses "assigned female at birth (AFAB)" as its primary population descriptor, which is appropriate for capturing the intended biological inclusion criterion. However, the application of this terminology is inconsistent and at times internally contradictory. On page 3 of the protocol, participants are described as "women assigned female gender at birth (AFAB), reflecting female biological sex." This conflates gender assignment at birth with biological sex in a way that is terminologically inaccurate AFAB refers to sex assigned at birth, not gender assigned at birth, and it reflects social designation rather than biological sex per se. On page 7 of the eligibility criteria, the phrasing shifts to "women assigned female at birth (AFAB), reflecting female biological gender" here "biological gender" is used, which is not a standard term. In the exclusion criteria, studies are to be excluded if "the study population includes transgenders that were not AFAB and not of female biological gender at birth" this phrasing is both grammatically incorrect ("transgenders" is not standard usage; "transgender people" or "transgender individuals" is preferred) and logically redundant given the inclusion criterion. The authors should consult current guidelines on inclusive and accurate sex/gender terminology and apply a consistent, precise, and respectful framework throughout.

5. The PRISMA flow diagram (Supplementary File 2) contains unresolved placeholder text and is incomplete.

The submitted PRISMA flow diagram includes placeholder entries such as "Reason 1 (n =)," "Reason 2 (n =)," and "Studies included in review (n = 134 + xxx)." The figure appears to have been partially populated with real data at some stages (e.g., 529 full-text articles assessed) but remains incomplete at others. While it is acknowledged that this is an ongoing review, submitting a PRISMA diagram with "xxx" placeholders and blank exclusion reason counts is not appropriate for a peer-reviewed publication, even at the protocol stage. The diagram should reflect the current known state of the review accurately and completely, with a clear note indicating which elements remain in progress. The notation "n = 134 + xxx" is particularly problematic, as it implies some included studies have been identified but the number is unknown this should be clarified.

6. The rationale for the 1 February 2018 start date relative to the prior Smit et al. (2019) review requires verification.

The authors state that the 1 February 2018 start date "reflects the endpoint of the prior meta-synthesis by Smit et al. (2019)." However, Smit et al. (2019) was published in Social Science & Medicine in 2019 , it is not immediately clear that February 2018 precisely corresponds to the search cutoff of that review rather than its publication date. The authors should verify and state explicitly what the search end date of the Smit et al. (2019) review was, and confirm that the chosen start date avoids both gap and overlap with the prior synthesis. If there is a temporal gap between the end of the previous search and the start of this one, the potential for missing studies in that interval should be acknowledged.

MINOR COMMENTS

7. The abstract omits the search date range and does not mention the OSF registration number. For a protocol abstract, readers need to be able to locate the registration and understand the temporal scope of the search at a glance. Both the OSF registration link (https://osf.io/wgzck/) and the date range (1 February 2018 – 31 January 2026) should appear in the abstract.

8. The "Strengths and Limitations" section lists only one limitation. Restricting to English-language peer-reviewed publications is acknowledged, but several other notable limitations are not mentioned here (though some appear elsewhere in the manuscript), including: the absence of grey literature and dissertations/theses, the PROSPERO non-registration, and the restriction to a single synthesis approach (meta-aggregation), which precludes interpretive or critical synthesis of findings. A more complete limitations box would strengthen the protocol's transparency.

9. There is a typographic error in the Introduction. On page 4 (line 99), "develoing countries" should read "developing countries."

10. The role of the guarantor is incorrectly assigned. The footnote states that "KEO serves as the guarantor of the review." However, the corresponding author is SDM (Seemela Desiree Malope). If the guarantor and corresponding author are different individuals, this should be

explicitly clarified. Typically, the corresponding author assumes guarantor responsibilities unless otherwise justified.

11. The data extraction process is described twice with slightly different details. The Data Extraction section states that extraction will be undertaken using "a structured form developed and piloted within Covidence" and then immediately restates that extraction will use "a structured data extraction form developed and piloted by the review team in Microsoft Excel." This reads as a duplication of two slightly different processes and should be reconciled into a single, clear description of the extraction workflow and tools.

12. The search strategy does not mention grey literature or hand-searching. The exclusion of grey literature, dissertations, and conference abstracts is explicitly stated in the exclusion criteria, but the protocol does not mention whether reference lists of included studies will be hand-searched, or whether any citation tracking (forward or backward) will be undertaken. JBI qualitative review guidance recommends reference list checking as a standard third step in the search process. If this is planned, it should be described; if not, the omission should be justified.

13. The ConQual table description could be strengthened. The protocol commits to producing a ConQual summary of findings table, which is appropriate. However, it does not describe how downgrading decisions will be made (e.g., under what circumstances dependability or credibility will be rated down), nor whether all reviewers will independently complete ConQual assessments before discussion. Providing this level of detail would enhance reproducibility.

14. Minor referencing inconsistencies. The in-text citation style shifts between author-date (e.g., "Smit et al., 2019") and a format with semicolons between sources in parentheses. One citation "Lockwood C et al., 2024" does not follow the author-date format used elsewhere (it uses an initial). The reference for Gripsrud (2008) on page 4 is incomplete, lacking a title, journal, and page numbers (it lists only chapter pages "31–44" with no book title). These should be corrected.

15. The phrase "the literature is saturated with studies" on page 4 is informal and imprecise for an academic manuscript. Suggested revision: "A substantial body of literature documents the risk factors, clinical features, treatment options, and physical sequelae of breast cancer."

DECISION

Minor to Moderate Revision Required

This protocol addresses a relevant and timely question and is grounded in a well-established methodological framework. The team's expertise in JBI qualitative synthesis is evident. However, the manuscript requires revision before acceptance. The most pressing issues are: the need for a more transparent and critical discussion of the PROSPERO non-registration; the reconciliation of the PICo/SPIDER framework duplication; the resolution of discrepancies between the databases listed in the text and those actually searched; the correction of sex/gender terminology; and the cleaning of the PRISMA flow diagram. The minor issues typographic errors, referencing inconsistencies, and the incomplete limitations box are straightforward to address.

None of the issues identified represent fundamental flaws in the review's scientific design or render the protocol unpublishable. The authors are encouraged to address all points raised above in their revision. If the registration and search strategy inconsistencies are fully resolved, this protocol has the potential to serve as a useful foundation for an important and impactful qualitative synthesis.

VERSION 1 - AUTHOR RESPONSE

MAJOR COMMENTS

1. The PROSPERO non-registration is a significant transparency concern that requires more direct acknowledgment. The authors state that the review was initially intended for PROSPERO registration but that searches had already commenced when registration was sought, making the protocol ineligible. This is disclosed which is appreciated but it is presented rather neutrally, without acknowledgment of the implications. PROSPERO registration prior to searching is a fundamental transparency safeguard in systematic review methodology, designed precisely to prevent post-hoc modification of eligibility criteria, outcomes, and analytic decisions based on knowledge of the literature. The fact that searching began before registration was secured represents a protocol deviation that could compromise the credibility of the review. The authors should: (a) explain how and why searching commenced prior to registration; (b) confirm explicitly that eligibility criteria, search strategy, and synthesis approach were finalized before any screening or extraction decisions were made; (c) acknowledge this as a limitation of the protocol's transparency rather than presenting it as a straightforward administrative alternative. Simply registering on OSF does not fully mitigate the loss of the pre-search time-stamp that PROSPERO would have provided.

1. Response to reviewers:

We thank the reviewer for this important comment and here are our feedback

a) As clarified during the earlier editorial query, the literature search was completed in January 2026 while the review was still ongoing. The review team initially intended to register the protocol with PROSPERO; however, the registration enquiry was made after database searching had already commenced and was nearing completion. This occurred because the team proceeded with the database search after agreeing on the review question, eligibility criteria, search strategy and planned JBI meta-aggregation approach, while the protocol manuscript was still being finalised for submission.

b) We confirm that the eligibility criteria, search strategy and planned JBI meta-aggregation synthesis approach were specified before screening, data extraction and synthesis decisions were undertaken and were not modified in response to emerging findings from the literature.

c) We acknowledge that the absence of PROSPERO registration before commencement of the search is a limitation of the protocol's transparency, as the review does not have a pre-search PROSPERO timestamp. The protocol was subsequently registered on OSF while the review was ongoing to provide a publicly accessible, time-stamped record of the review methodology and update process. We have revised the Registration and Strengths and limitations sections to acknowledge this more explicitly.

2. The manuscript describes two different eligibility frameworks (PICo and SPIDER) without adequately reconciling them. The abstract and introduction state that the review is "guided by the PICo framework (Population, Interest, Context)." The eligibility criteria section then states that "Eligibility criteria were defined a priori using a modified SPIDER framework (Sample, Phenomenon of Interest, Design, Evaluation, Research type)." These two frameworks serve overlapping but distinct purposes, and their simultaneous use without explanation will confuse readers. PICo is appropriate for framing the review question, while SPIDER is more appropriate for operationalising eligibility criteria in qualitative reviews but this distinction is never explained. The authors should clarify how the two frameworks were used at different stages of the review process, or consolidate the framing to use one primary framework consistently throughout.

2. Response to reviewers:

We thank the reviewer for this helpful comment. We agree that the relationship between PICo and SPIDER required clearer explanation. We have revised the manuscript to clarify that PICo was used to frame the overarching review question, while the modified SPIDER framework was used to operationalise the eligibility criteria for study selection. PICo defined the population, phenomenon of interest and context of the review, whereas SPIDER provided additional specificity for this qualitative evidence synthesis by specifying the sample, qualitative study designs, evaluation of experiential accounts and research type. The two frameworks were therefore applied at different stages of the review process and were not used interchangeably

3. The search strategy is inconsistently described across the manuscript. The methods section states that the following databases will be searched: "Web of Science, Scopus, PubMed, MEDLINE, and EBSCOhost platform" listing five databases, and earlier the abstract adds

"ProQuest" and "ScienceDirect," implying six or seven sources. However, Supplementary File 1 (the actual search table) documents searches in only four databases: PubMed, EBSCOhost/CINAHL, Scopus, and Web of Science with no search conducted in ProQuest, ScienceDirect, or MEDLINE as a separate database. The PRISMA flow diagram confirms that 11,649 records were identified from four databases. This discrepancy between what the text promises and what was actually done must be resolved. If ProQuest and ScienceDirect were dropped, the rationale must be provided. Additionally, the search strings across the four databases are not parallel for example, the Web of Science string omits several experiential terms ("illness perception," "coping strateg*," "emotional impact") that are included in the PubMed and CINAHL strings. This inconsistency should be explained or corrected to ensure sensitivity is equivalent across databases.

3. Response to reviewers:

We agree and have corrected the manuscript to align the Methods section, abstract, PRISMA information and supplementary search table. The text now reports only the databases that were searched: PubMed, EBSCOhost/CINAHL, Scopus and Web of Science. References to ProQuest, ScienceDirect and MEDLINE as a separate database have been removed. MEDLINE-indexed records were accessed through PubMed rather than searched as a separate database. We have also clarified that search strings were adapted to the syntax, indexing and functionality of each database while retaining the same core search concepts: breast cancer, lived experience/experiential accounts, and qualitative research methods.

4. The gender and sex terminology is inconsistently and in places incorrectly applied. 4.1. The manuscript uses "assigned female at birth (AFAB)" as its primary population descriptor, which is appropriate for capturing the intended biological inclusion criterion. However, the application of this terminology is inconsistent and at times internally contradictory. On page 3 of the protocol, participants are described as "women assigned female gender at birth (AFAB), reflecting female biological sex." This conflates gender assignment at birth with biological sex in a way that is terminologically inaccurate AFAB refers to sex assigned at birth, not gender assigned at birth, and it reflects social designation rather than biological sex per se. On page 7 of the eligibility criteria, the phrasing shifts to "women assigned female at birth (AFAB), reflecting female biological gender" here "biological gender" is used, which is not a standard term. **4.2.** In the exclusion criteria, studies are to be excluded if "the study population includes transgenders that were not AFAB and not of female biological gender at birth" this phrasing is both grammatically

incorrect ("transgenders" is not standard usage; "transgender people" or "transgender individuals" is preferred) and logically redundant given the inclusion criterion. The authors should consult current guidelines on inclusive and accurate sex/gender terminology and apply a consistent, precise, and respectful framework throughout.

4. Response to reviewers:

4.1. We thank the reviewer for identifying this inconsistency. We agree that the original wording incorrectly conflated sex assigned at birth, biological sex, and gender. We have revised the manuscript throughout to use accurate and consistent terminology, replacing the incorrect references to "gender assigned at birth" and "biological gender" with "sex assigned at birth," where appropriate. The eligibility criteria and all relevant sections now consistently refer to **sex assigned at birth**, ensuring that the terminology accurately reflects the intended study population and aligns with current scientific and reporting standards.

4.2. We sincerely thank the reviewer for identifying this issue and apologize for the inappropriate and imprecise terminology used in the original manuscript. We agree that the term “transgenders” is not appropriate usage and that “transgender individuals” is the correct, respectful, and clinically appropriate terminology. We have revised the exclusion criterion accordingly to replace “transgenders” with “transgender individuals.”

We also acknowledge the reviewer’s point regarding the need for consistency and precision in sex and gender terminology. The wording has been carefully revised to ensure that the eligibility criteria accurately reflect the intended study population and align with contemporary recommendations for inclusive and respectful reporting of sex and gender-related information. We have reviewed the manuscript to ensure that terminology is applied consistently throughout.

5. The PRISMA flow diagram (Supplementary File 2) contains unresolved placeholder text and is incomplete. The submitted PRISMA flow diagram includes placeholder entries such as "Reason 1 (n =)," "Reason 2 (n =)," and "Studies included in review (n = 134 + xxx)." The figure appears to have been partially populated with real data at some stages (e.g., 529 full-text articles assessed) but remains incomplete at others. While it is acknowledged that this is an ongoing review, submitting a PRISMA diagram with "xxx" placeholders and blank exclusion reason counts is not appropriate for a peer-reviewed publication, even at the protocol stage. The diagram should reflect the current known state of the review accurately and completely, with a clear note indicating which elements remain in progress. The notation "n = 134 + xxx" is particularly

problematic, as it implies some included studies have been identified but the number is unknown this should be clarified.

5. Response to reviewers:

We thank the reviewer for highlighting this issue. We have revised Supplementary File 2 to remove unresolved placeholders and to present the current review status accurately. As this is a protocol submission and full-text eligibility assessment is ongoing, the final number of included studies and full-text exclusion reasons are not yet available. The PRISMA flow diagram has therefore been revised to show the completed stages to date, including records identified, duplicates removed, records screened, records excluded at title/abstract stage, and reports assessed for eligibility. A note has been added to indicate that the final included study count and full-text exclusion reasons will be reported in the completed systematic review.

6. The rationale for the 1 February 2018 start date relative to the prior Smit et al. (2019) review requires verification. The authors state that the 1 February 2018 start date "reflects the endpoint of the prior meta-synthesis by Smit et al. (2019)." However, Smit et al. (2019) was published in Social Science & Medicine in 2019, it is not immediately clear that February 2018 precisely corresponds to the search cutoff of that review rather than its publication date. The authors should verify and state explicitly what the search end date of the Smit et al. (2019) review was and confirm that the chosen start date avoids both gap and overlap with the prior synthesis. If there is a temporal gap between the end of the previous search and the start of this one, the potential for missing studies in that interval should be acknowledged. previous search and the start of this one, the potential for missing studies in that interval should be acknowledged.

6. Response to reviewers:

Thank you for this important observation. We have verified the search strategy reported in the supplementary materials of Smit et al. (2019). The review searched the literature from 1988 through January 2018, with the search concluding in January 2018. Consequently, we selected 1 February 2018 as the start date for our search to ensure temporal continuity with the previous review while avoiding duplication of studies already captured by Smit et al. (2019).

We have revised the manuscript to explicitly state that the 1 February 2018 start date was chosen because it immediately follows the search end date of the previous systematic review, rather than its publication date. This clarification confirms that there is no

temporal gap or overlap between the evidence syntheses and that studies published after the completion of the Smit et al. search were eligible for inclusion in the present review.

The relevant text has been amended in the Methods section to read as follows:

"The search commenced on 1 February 2018, immediately following the January 2018 search end date of the previous systematic review by Smit et al. (2019), thereby ensuring continuity of the evidence base while avoiding overlap in study inclusion."

MINOR COMMENTS

7. The abstract omits the search date range and does not mention the OSF registration number. For a protocol abstract, readers need to be able to locate the registration and understand the temporal scope of the search at a glance. Both the OSF registration link (https://osf.io/wgzck/) and the date range (1 February 2018 – 31 January 2026) should appear in the abstract.

7. Response to reviewers: The date range and OSF link have been integrated in the abstract as follows: "This review updates prior evidence by synthesising studies published between 1 February 2018 – 31 January 2026 and has been prospectively registered on OSF (https://osf.io/wgzck/)."

8. The "Strengths and Limitations" section lists only one limitation. Restricting to English-language peer-reviewed publications is acknowledged, but several other notable limitations are not mentioned here (though some appear elsewhere in the manuscript), including: the absence of grey literature and dissertations/theses, the PROSPERO non-registration, and the restriction to a single synthesis approach (metaaggregation), which precludes interpretive or critical synthesis of findings. A more complete limitations box would strengthen the protocol's transparency.

8. Response to reviewers: We have expanded the study limitations to read as follow:
Limitations

- Restriction to English-language, peer-reviewed publications may limit representation of non-English and unpublished qualitative evidence
- The search strategy will be restricted to peer-reviewed published literature and excluded grey literature (eg, technical reports, policy documents, conference proceedings and preprints) as well as dissertations and theses. This may result in the omission of relevant evidence, increased the risk of publication bias, and limited the comprehensiveness of the review.

- Although the review protocol was prospectively registered on the Open Science Framework (OSF), it was not registered in PROSPERO. This may reduce the visibility and discoverability of the protocol among researchers who routinely search PROSPERO for prospectively registered systematic reviews.
- The review will employ meta-aggregation as the sole qualitative synthesis approach. While this method provides a rigorous and practice-oriented synthesis that remains closely aligned with the primary studies, it precludes more interpretive or critical forms of synthesis, limiting opportunities for theory generation, conceptual development, and deeper exploration of contextual and explanatory insights.

9. There is a typographic error in the Introduction. On page 4 (line 99), "develoing countries" should read "developing countries."

9. Response to reviewers: Typographic error in "develoing countries" has been rectified to "developing countries."

10. The role of the guarantor is incorrectly assigned. The footnote states that "KEO serves as the guarantor of the review." However, the corresponding author is SDM (Seemela Desiree Malope). If the guarantor and corresponding author are different individuals, this should be explicitly clarified. Typically, the corresponding author assumes guarantor responsibilities unless otherwise justified.

10. Response to reviewers: At the time of study conception, KEO was intended to be the guarantor. However, the authors subsequently agreed that the first author (SDM) should serve as the guarantor. This has now been corrected in the manuscript, with the first author appropriately designated as the guarantor and corresponding author. The correction reads as follows: "SDM serves as the guarantor of the review and takes responsibility for the integrity of the work".

11. The data extraction process is described twice with slightly different details.

The Data Extraction section states that extraction will be undertaken using "a structured form developed and piloted within Covidence" and then immediately restates that extraction will use "a structured data extraction form developed and piloted by the review team in Microsoft Excel." This reads as a duplication of two slightly different processes and should be reconciled into a single, clear description of the extraction workflow and tools.

11. Response to reviewers: The statement has been revised from **"**Data extraction will be undertaken using a structured form developed and piloted within Covidence. Qualitative data will be extracted independently by two reviewers (KEO and SDM) using a structured data extraction form developed and piloted by the review team in Microsoft Excel." to "Data extraction will be undertaken independently by two reviewers (SDM and KEO) using a structured data extraction form developed and piloted within Covidence. Following extraction, the data will be exported to a standardized Microsoft Excel spreadsheet for further data management, verification, and analysis. The data extraction form will be refined, where necessary, following pilot testing to ensure consistency, completeness, and applicability across the included studies. Any discrepancies between reviewers will be resolved through discussion and, where required, consultation with a third reviewer."

12. The search strategy does not mention grey literature or hand-searching.

The exclusion of grey literature, dissertations, and conference abstracts is explicitly stated in the exclusion criteria, but the protocol does not mention whether reference lists of included studies will be hand-searched, or whether any citation tracking (forward or backward) will be undertaken. JBI qualitative review guidance recommends reference list checking as a standard third step in the search process. If this is planned, it should be described; if not, the omission should be justified.

12. Response to reviewers: We will not include grey literature, dissertations, or conference abstracts in our search strategy. We also will not undertake hand-searching of journals or forward/backward citation tracking. However, we will perform reference list checking of all included studies to identify any additional potentially eligible articles, consistent with the third stage of the JBI qualitative evidence synthesis search strategy. We have revised the manuscript to explicitly describe this step in the search methods to improve transparency and reproducibility.

13. The ConQual table description could be strengthened. The protocol commits to producing a ConQual summary of findings table, which is appropriate. However, it does not describe how downgrading decisions will be made (e.g., under what circumstances dependability or credibility will be rated down), nor whether all reviewers will independently complete ConQual assessments before discussion. Providing this level of detail would enhance reproducibility.

13. Response to reviewers:

We thank the reviewer for this helpful comment. We have strengthened the ConQual section to clarify how confidence assessments and downgrading decisions will be undertaken. The revised manuscript now states that two reviewers (KEO and SDM) will independently complete ConQual assessments before discussion and consensus, with unresolved disagreements resolved through review team discussion. We have also clarified that each synthesised finding will initially be considered high confidence and may be downgraded based on concerns relating to dependability and/or credibility. Downgrading decisions and justifications will be documented in the ConQual summary of findings table in the completed review.

14. Minor referencing inconsistencies. The in-text citation style shifts between author-date (e.g., "Smit et al., 2019") and a format with semicolons between sources in parentheses. One citation "Lockwood C et al., 2024" does not follow the authordate format used elsewhere (it uses an initial). The reference for Gripsrud (2008) on page 4 is incomplete, lacking a title, journal, and page numbers (it lists only chapter pages "31–44" with no book title). These should be corrected.

14. Response to reviewers:

"Lockwood C et al., 2024" has been correctly updated to "(Aromataris, 2024)"

"Gripsrud (2008)" has been correctly updated, the complete reference is Gripsrud, B. (2008). The Cultural History of the Breast. In I. V. Pitts (Ed.), *Cultural Encyclopedia of the Body* (Vol. 1, pp. 31-44). Greenwood Publishing

15. The phrase "the literature is saturated with studies" on page 4 is informal and imprecise for an academic manuscript. Suggested revision: "A substantial body of literature documents the risk factors, clinical features, treatment options, and physical sequelae of breast cancer."

15. Response to reviewers: We have removed the sentence "The literature is saturated with studies documenting the various breast cancer risk factors along with the disease's signs and symptoms, treatment, and management options for the disease and, the physical implications resulting from said treatment and management options" and replaced it with "A substantial body of literature documents the risk factors, clinical features, treatment options, and physical sequelae of breast cancer."
