# Supplementary material for: Exploring the lived experiences of women with breast cancer across the care continuum: a systematic meta-aggregative qualitative review protocol
Source: BMJ Open. 2026 Oct 20;16(10):e120045. doi: 10.1136/bmjopen-2026-120045 (PMC13684198; doi:10.1136/bmjopen-2026-120045)
Supplement: Supplementary file 2 [file bmjopen-16-10-s001.docx]

**Supplementary file 1**

| **Database** | **Platform** | **Search query** | **Date searched** | **Date limits applied** | **Language** | **Records retrieved** |
| --- | --- | --- | --- | --- | --- | --- |
| PubMed | NCBI | (“breast neoplasms”[MeSH Terms] OR “breast cancer”[Title/Abstract] OR “breast neoplasm*”[Title/Abstract] OR “breast carcinoma*”[Title/Abstract] OR “breast tumor*”[Title/Abstract]) AND (“lived experience*” OR “patient narrative*” OR “patient perspective*” OR survivorship OR “emotional impact” OR “coping strateg*” OR “social support” OR “psychological aspect*” OR “quality of life” OR “illness perception” OR “treatment experience*” OR “patient journey*” OR stigma OR barrier* OR pathway* OR enabler* OR facilitator*) AND (qualitative OR interview* OR “focus group*” OR ethnograph* OR phenomenolog* OR “grounded theory”) | **February 2026** | **1 Feb 2018 – 31 Jan 2026** | English | **1,844** |
| EBSCOhost / CINAHL | EBSCO | (breast neoplasm* OR breast cancer OR breast carcinoma OR breast tumor) AND (“lived experience*” OR “patient narrative*” OR “patient perspective*” OR survivorship OR “emotional impact” OR “coping strateg*” OR “social support” OR “psychological aspect*” OR “quality of life” OR “illness perception” OR “treatment experience*” OR “patient journey*” OR stigma OR barrier* OR pathway* OR enabler* OR facilitator*) AND (qualitative OR ethnograph* OR phenomenolog* OR interview* OR “focus group*”) | **February 2026** | **1 Feb 2018 – 31 Jan 2026** | English | **8,702** |
| Scopus | Elsevier | TITLE-ABS-KEY (“breast cancer” OR “breast neoplasm*” OR “breast carcinoma” OR “breast tumor”) AND TITLE-ABS-KEY (“lived experience*” OR “patient narrative*” OR “patient perspective*” OR survivorship OR “emotional impact” OR “coping strateg*” OR “social support” OR “quality of life” OR “treatment experience*” OR “patient journey*” OR stigma OR barrier* OR pathway* OR facilitator*) AND TITLE-ABS-KEY (qualitative OR ethnograph* OR phenomenolog* OR interview* OR “focus group*”) | **February 2026** | **2018–2026** | English | **162** |
| Web of Science | Clarivate | TS=(“breast cancer” OR “breast neoplasm*” OR “breast carcinoma” OR “breast tumor*”) AND TS=(“lived experience*” OR “patient narrative*” OR “patient perspective*” OR “patient journey*” OR survivorship OR “quality of life” OR “treatment experience*” OR stigma OR barrier* OR pathway*) AND TS=(qualitative OR ethnograph* OR phenomenolog*) | **February 2026** | **1 Feb 2018 – 31 Jan 2026** | English | 941 |

**Total records identified across all sources - 11,649**
