# Supplementary material for: Exploring the lived experiences of women with breast cancer across the care continuum: a systematic meta-aggregative qualitative review protocol
Source: BMJ Open. 2026 Oct 20;16(10):e120045. doi: 10.1136/bmjopen-2026-120045 (PMC13684198; doi:10.1136/bmjopen-2026-120045)
Supplement: Supplementary file 3 [file bmjopen-16-10-s002.docx]

**Supplementary file 2 - Prisma flow so far**

**Identification of studies via databases and registers**

Records identified from*:

**Databases (n = 11,649)**

PubMed (n = 1,844)

EBSCOhost / CINAHL (n = 8,702)

Scopus (n = 162)

Web of Science (n = 941)

Records removed *before screening*:

Duplicate records removed

(n = 6276)

**Identification**

**Records screened**

**(n = 5373)**

Records excluded**

(n = 4844)

Reports sought for retrieval

(n = 529)

Reports not retrieved

(n = 0)

**Screening**

Reports assessed for eligibility

Full article screening (n = 529)

Reports excluded:

( n= number with reasons to be reported in completed review)

Studies included in review

(n = final number to be reported i

n the completed systematic review)

**Included**

*Consider, if feasible to do so, reporting the number of records identified from each database or register searched (rather than the total number across all databases/registers).

**If automation tools were used, indicate how many records were excluded by a human and how many were excluded by automation tools.

Source: Page MJ, et al. BMJ 2021;372:n71. doi: 10.1136/bmj.n71.

This work is licensed under CC BY 4.0. To view a copy of this license, visit <https://creativecommons.org/licenses/by/4.0/>
